# “This Is Going To Be Different, But It's Not Impossible”: Adapting To Telehealth Occupational Therapy For Autistic Children

**DOI:** 10.5195/ijt.2024.6608

**Published:** 2024-06-28

**Authors:** Amber M. Angell, Elinor E. Taylor, Joana Nana Serwaa Akrofi, Elaine D. Carreon, Marshae D. Franklin, Julie Miller, Catherine Crowley, Shona Orfirer Maher

**Affiliations:** 1 USC Chan Division of Occupational Science and Occupational Therapy, University of Southern California, Los Angeles, California, USA; 2 Department of Occupational Therapy, New York University, New York, New York, USA; 3 Nurture Collective, Whittier, California, USA; 4 Professional Child Development Associates, Pasadena, California, USA; 5 Devoted Educational Solutions, Los Angeles, California, USA

**Keywords:** Autism, COVID-19, Occupational therapy, Pandemic, Telehealth, Telerehabilitation

## Abstract

This qualitative study examined participants' experiences of transitioning to telehealth-delivered pediatric occupational therapy for autistic children during the COVID-19 pandemic. We interviewed three clinic administrators, four occupational therapists, and six parents of autistic children (*n*=13) from three Los Angeles area clinics over a 7-month period. Our narrative and thematic analyses yielded three overarching themes: *Transformative Experiences*, *Reimagining Therapy*, and *Going Forward*. Overall, we found that the transition to telehealth shifted participants' preconceptions about themselves, their relationships, and the nature of occupational therapy. Many deepened their relationships; adapted interventions; uncovered surprising capabilities; and challenged the occupational therapy status quo to advocate for ongoing virtual delivery. Our findings bear relevance to the body of current literature debating the post-pandemic viability of telehealth-delivered occupational therapy.

Occupational therapy has traditionally been conducted in-person (Camden & Silva, 2021). However, the COVID-19 pandemic provided novel opportunities to explore how occupational therapy could be implemented virtually through telehealth (Ranjan et al., 2023). Prior to the pandemic, telehealth delivery was relatively rare in occupational therapy (Camden & Silva, 2021; Ranjan et al., 2023). Much of the existent pre-pandemic literature focused on telehealth as a *supplement* to in-person services (Gibbs & Toth-Cohen, 2011). The pandemic, however, necessitated widespread transition to telehealth service delivery. This unique circumstance created a kind of ‘natural experiment,’ an opportunity for research to examine the extent to which telehealth-delivered occupational therapy could be implemented effectively in *fully* virtual formats, and what impacted acceptability by clients, families, and providers.

In the years following the onset of COVID-19, a number of studies drew from this ‘natural experiment’ to establish new findings pertaining to telehealth-delivered occupational therapy. In a scoping review, Ranjan et al. (2023) found that the pandemic forced occupational therapists to rapidly change the status quo of in-person delivery – a process that entailed significant barriers and required extensive adaptations. An *OTJR: Occupational Therapy Journal of Research* special issue on telehealth similarly noted that telehealth went from an ‘emerging’ delivery model to defining everyday practice during COVID-19 (Little & Proffitt, 2023). Studies included within this special issue broadly concluded that telehealth-delivered occupational therapy presents both advantages and limitations across varying practice settings and populations (Angell et al., 2023; Lamash et al., 2023; Little et al., 2023; Pineda et al., 2023; Sleight et al., 2023). Participants from these studies often supported ongoing telehealth-delivered occupational therapy, but not necessarily as a universal replacement for in-person care (Angell et al., 2023; Pineda et al., 2023). Findings further indicated that telehealth may be particularly effective for certain intervention types (e.g., providing education or parent coaching) (Angell et al., 2023; Little & Proffitt, 2023; Sleight et al., 2023), or as an option for occupational therapists, clients, and families who experience barriers in providing, accessing, or engaging with in-person services (Angell et al., 2023; Lamash et al., 2023; Pineda et al., 2023; Sleight et al., 2023).

Even prior to COVID-19, telehealth had been identified as a promising delivery method of clinical services for autistic clients in particular (Ashburner et al., 2016; Gibbs & Toth-Cohen, 2011; Little et al., 2023; Little, Pope, et al., 2018; Little, Wallisch, et al., 2018). Autistic participants have often (although not universally) endorsed a preference for telehealth over in-person healthcare services (Lamash et al., 2023). In a systematic review, Ellison et al. (2021) reported that telehealth services were at least equivalent to, if not better than, in-person services for autistic people and their families. These pre-pandemic findings are particularly salient given growing demands for autism services that are accessible, strengths-based, and reduce disparities in access to care (Angell et al., 2018; Dallman et al., 2022; Taylor, 2022).

The pandemic provided further opportunities for researchers to examine the efficacy and perceptions of telehealth-delivered occupational therapy for autistic clients. Lamash et al. (2023) suggested that telehealth could promote access to strengths-based interventions for autistic clients, while Little et al. (2023) found that their telehealth-delivered occupational therapy intervention led to increases in toileting skills for autistic children, as well as parental satisfaction with those skills. They noted that synchronous videoconferencing sessions, in which practitioners provided live, real-time education and reflective coaching aimed at empowering parents to support their children's toilet training, were a particularly effective component of this intervention. Rosenfeld and Brooks (2023) found that pandemic-era telehealth-delivered occupational therapy demanded creativity from occupational therapists, reduced social anxiety for autistic participants by situating therapy in the home environment, and “compelled and empowered families to become involved in their [autistic] child's therapy” (p. 8). Camden and Silva (2021) argued that, following the pandemic, telehealth should be offered as an ongoing delivery option to increase the accessibility, cost-effectiveness, and family-centeredness of occupational therapy services for families of children with disabilities. However, they cautioned against a one-size-fits-all model, noting that a hybrid approach may be best for some therapists and clients.

These findings bear overlap with the results of our prior work (Angell et al., 2023). We found that clinic administrators, occupational therapists, and parents of autistic children faced barriers and facilitators to the telehealth transition during the pandemic, as well as positive and negative therapeutic outcomes. However, the iterative nature of qualitative research, which enables researchers to follow additional threads that arise in the data, led us to explore unexpected findings related to participants' lived experiences. We therefore, in this paper, report findings from a subcorpus of the larger dataset, asking: *How did the participants experience the transition to telehealth-delivered occupational therapy services?* In asking this question, we endeavor to capture the richness of participants' experiences, including how they narrated the emotional landscape of the telehealth transition, and how, despite challenges, some had unexpected (e.g., ‘transformative') experiences. In doing so, we aim to contribute to the burgeoning body of research on telehealth-delivered occupational therapy, by providing a rich, nuanced, narrative, emic (i.e., experience-near) understanding of the transition to telehealth-delivered occupational therapy, highlighting the complex, diverse, and often transformative nature of their journeys in adapting to telehealth in the context of the pandemic.

## Methods

In this qualitative cross-sectional study, we aimed to understand the experiences of the sudden transition from in-person to telehealth-delivered occupational therapy services at the outset of the COVID-19 pandemic. The University of Southern California Institutional Review Board approved this study.

### Recruitment

Our research design aimed to capture multiple perspectives. We therefore recruited three types of participants who experienced the transition to telehealth: Clinic administrators, occupational therapists, and parents/caregivers of autistic children receiving occupational therapy services. We used purposive sampling to recruit from three Los Angeles County community-based pediatric clinics where occupational therapy services are provided, selecting clinics that (primarily) used different occupational therapy practice models: (1) the Developmental, Individual-differences, and Relationship based (DIR®)/Floortime™ Model (Greenspan & Wieder, 2008), (2) Ayres Sensory Integration® (Ayres, 1972), and (3) the Early Start Denver Model (ESDM; Rogers, 2016). Clinic administrators distributed electronic flyers to eligible occupational therapists and parents/caregivers of autistic children. Prospective participants contacted the study team by email or phone. Inclusion criteria were: (1) a clinic administrator, occupational therapist, or parent/caregiver of an autistic child; (2) at least 18 years of age; and (3) able to participate in an English-language interview. All participants provided written consent using an IRB-approved online platform.

**Table 1:** Participant Characteristics

Clinic	Administrators*	Occupational Therapists	Parents	Total by Clinic
1	1	4	1	6
2	1	1	3	5
3	1	1	0	2
**Total by Participant Type**	3	6	4	13

* *Note.* All administrators were also registered occupational therapists.

### Data Collection

We used Zoom to conduct semi-structured interviews with 13 participants (three administrators, six occupational therapists, and four parents) lasting 60-90 minutes each. Interviews took place between April and November 2021. We developed semi-structured interview guides for each participant group that utilized a narrative approach, (i.e., aiming to elicit stories). Each participant received an e-gift card after participating in the interview. Audio-recorded interviews were transcribed verbatim for analysis, replacing all identifiers with pseudonyms.

### Data Analysis

We used narrative and thematic analyses (Braun & Clarke, 2006; Reissman, 1993) to code the interviews. First, two authors read six transcripts (i.e., one from each study site) to develop an initial codebook. Five authors then coded the data corpus using NVivo 12; each transcript was coded by two authors. The study team met throughout the coding process to discuss and refine the codebook. Here we report findings from a data subcorpus focused on descriptive and emic perspectives on the telehealth transition; another subcorpus from the larger dataset, focused on barriers and facilitators, has been published elsewhere (Angell et al., 2023).

### Trustworthiness

To build credibility and trustworthiness (Letts et al., 2007), we incorporated multiple perspectives into our data by examining the experience of the telehealth transition from three different participant types. Within our team, we incorporated multiple perspectives in our analytic process by including investigators from diverse disciplinary backgrounds, racial/ethnic groups, nationalities, and neurotypes. Two authors are clinic administrators who experienced the transition to telehealth during the pandemic and contributed to analysis and interpretation, further enhancing credibility and confirmability.

## Results

Our analysis yielded three main themes related to the participants' experiences of the transition and adjustment to telehealth: *Transformative Experiences*, *Reimagining Therapy*, and *Going Forward*. In addition to data excerpts below that illustrate the themes and subthemes, we have also selected four vignettes (i.e., descriptive narratives of particular participants' experiences) as examples of the nuanced complexities and range of emotional tone that were present throughout the data corpus.

### Transformative Experiences

We anticipated that participants would find the telehealth transition challenging in various ways; we did not expect that some of them would also describe the experience as transformative. Within this unexpected finding of *Transformative Experiences*, we identified three sub-themes: (1) *Transformed Therapeutic Facilitation*, (2) *Transformed Ways-Of-Being-Together*, and (3) *Personal Transformation.*

#### Transformed Therapeutic Facilitation

There was a consensus among participants about the crucial role of parents in facilitating telehealth occupational therapy sessions. We have reported elsewhere the factors that hindered or facilitated the transition to telehealth, which required significant parent involvement (Angell et al., 2023). Here, however, our analytic lens shifts to the *experiential* and *transformative* aspects of this phenomenon. Specifically, participants spoke about how the necessity of parent involvement in telehealth sessions transformed parent engagement in profound ways.

For Clinic 1 therapists and parents, parent coaching was a standard component of participants' pre-pandemic occupational therapy experiences, but for the other two clinics (and presumably for most occupational therapists), it was not. The transition to telehealth necessitated that all occupational therapists adopt parent coaching as a primary modality, shifting many responsibilities pertaining to facilitation of hands-on therapeutic activities from therapists to parents. Even occupational therapists experienced in parent coaching were challenged in transitioning to a *fully* remote, *solely* coaching-based delivery model. This change transformed interpersonal dynamics and the norms of occupational therapy practice, shifting the standard of care from therapist-directed to parent co-facilitated.

Parents described differences in occupational therapy sessions prior to versus during the pandemic. In prior occupational therapy sessions, generally speaking, parents could choose to attend their child's occupational therapy sessions, but they were not typically required to attend. (At Clinic 1, where a DIR®/Floortime™-based parent coaching model was utilized, parents were typically present in sessions, where therapists coached them to engage with their child; however, there were still many opportunities for parents to simply observe and allow the occupational therapist to direct the session.) Some parents described how the structure of pre-pandemic in-person sessions had hindered their ability to participate and get feedback about their child's therapy. Alexis (Clinic 1 parent) said, “I always want to be involved and a participant, but I also feel like a lot of these clinics^1^ didn't want the parents to participate. They just wanted you to kind of like, go away.” Fernando (Clinic 2 parent) reported that, with in-person occupational therapy, he had received few updates on how his son, Luis, was doing in therapy: “Before, it was all done at school, and we would get a report every six weeks or whatever.”

The shift to telehealth, however, transformed these norms. As therapists could not be in-person to facilitate interventions, parents were tasked with attending each session and taking on many of the therapeutic responsibilities and skills. Essentially, via telehealth, parents became a core locus of hands-on therapeutic facilitation. “Like we're of course guiding [parents], and then giving them these activities and suggestions,” Jenna (Clinic 1 occupational therapist) noted, “but yeah, they're the ones that are really doing all the beautiful, hard work.” Hannah (Clinic 3 occupational therapist) described how one parent she coached gradually evolved into taking on more of a facilitator role: “I think little by little, the mom developed confidence on how to have [her child] engage, based on the suggestions I was giving her.” Isabella (Clinic 1 occupational therapist) recounted how telehealth challenged parents and often helped them to better understand the rationale underlying interventions:

It's been really surprising to see the parents really step up in their ability to watch out for key things that, you know, a parent might usually miss because I'm working with the kid, or because, you know, they've just seen their child do it, and it's just never been, like, something that they attribute to, like, a sensory need, for example.

As a result of their increased engagement in therapy, many parents developed novel insights into occupational therapy processes and outcomes. Alexis said, “I went from being *told* [that concepts in occupational therapy were] important […] to now understanding, ‘*This* is what my son needs, and it's going to help him be a more functional person.'” Fernando appreciated how attending telehealth sessions with Luis allowed him to directly observe his son's progress: “You know, I don't know how we would have done if it had been in-person, but, like, I can definitely see him growing or him improving in certain areas. […] So being able to sit there with him has been really – was really good to see.” Zoe (Clinic 2 parent) emphasized that participating in her child's sessions was illuminating and allowed her to become comfortable with the flow of activities:

I actually learned stuff from participating. Because sometimes when you're at the clinic, you just actually observe, you know, you don't want to interrupt, like, the flow of the clinic. But like, it kind of helped, a lot, especially on the feeding part, like, you know, it's okay to make a mess. It's okay to let it go.

Mila (Clinic 2 parent), however, did not find the shift to be as helpful. Mila appreciated that in-person clinic services gave her the *choice* to attend sessions with her son, Enzio, as opposed to *requiring* her presence. When Mila did attend, she liked that she could focus on observing Enzio's occupational therapist, Lisa, as opposed to co-facilitating therapy. Mila further expressed that she preferred to observe in the clinic setting, as this allowed her to incorporate Lisa's techniques into her interactions with Enzio at her own pace and on her own schedule: “I just watched and learned and then did whatever she did. I copied her, whatever she did during the week. So, when it comes to participating, I didn't participate with them in clinic, but I did participate as a parent during the week.” Mila's experience conveys how in-person occupational therapy can provide greater flexibility and reduce stress for some parents.

Therapists described parental involvement as necessary for telehealth's success. While this necessity often bore positive results, it also meant that therapy sessions and outcomes depended on parents' availability, emotional regulation, and level of engagement with the coaching process. Jenna (Clinic 1 occupational therapist) described this:

Like if parents' regulation was just off, you know, that's going to impact our therapy sessions as well. So, if parents are having like just a really overwhelming day, they may not be as present or ready to engage. Versus in clinic, we would be able to be more hands-on, and kind of step in and help. In telehealth, we can't. So we're really needing that parent support and parent collaboration. So if parents aren't involved, I think that's the trickiest thing.

Therapists noted that parents experienced heightened stressors during the pandemic. Parents thus had to manage numerous demands that could make it difficult for them to engage in telehealth. Therapists often sought to understand and accommodate the extraordinarily stressful circumstances that many parents faced. “I had some parents that I think were just so overwhelmed with everything,” Lisa (Clinic 2 occupational therapist) recalled, “and I had to be careful because I just didn't want to throw more on top of them.” Hannah (Clinic 3 occupational therapist) said that parents' stress could come up during sessions; she consequently learned to shift from providing coaching to helping support these parents when needed. She recounted:

There were like so many sessions with [one] parent, where 90% of the time like she was just venting – like how, she's like, she did not anticipate what this was going to look like for her. […] I had to tell myself, it's okay, like, I'm not working on his goal right now. It's okay. Like, I'm here to support this parent.

Therapists and parents reported that therapeutic use of specific models impacted their adjustments to telehealth-based coaching. In particular, several participants noted that their transitions were made easier by the fact that Clinic 1 uniquely incorporated DIR®/Floortime™ into their occupational therapy practice. DIR®/Floortime™ is a model focused on client-parent emotional co-regulation, which often uses therapist-parent coaching to achieve this end (Greenspan & Wieder, 2008). Via this practice model, Clinic 1 therapists had experience prior to the pandemic with supporting parents' regulation and coaching parents to help conduct interventions. Jenna (Clinic 1 occupational therapist) said that this experience helped her adapt to the telehealth context, relative to other occupational therapists without the same specialized training. “I've been so thankful of working with Clinic 1 and […] how involved parents are in our sessions already,” she shared. “I heard from [occupational therapists at other clinics] that one of the hardest things for them was learning how to incorporate families into sessions.” Alexis (Clinic 1 parent) expressed that Clinic 1's prior use of parent coaching eased the stress of her telehealth transition:

Clinic 1, of course, they're all very parent coaching-oriented. They all work with small kids, and they see how important it is to have the parents with the child. And I feel like that was a strength in our case, because a lot of our therapists already feel comfortable coaching the parents. So [telehealth] didn't seem like it was as scary or as hard as I thought it was going to be.

Vignette #1 further illustrates this sub-theme, providing a nuanced look into how a Clinic 1 occupational therapist, Gabriela, went from being a telehealth novice, to adopting a fully virtual, parent coaching-based delivery model. Her story further contextualizes her therapeutic facilitation transformation against the background of the crises that the pandemic created for clinics. Her experiences also lend greater detail as *how* and *why* specific features of her Clinic 1 background subjectively translated to a telehealth context.

#### Vignette #1: Transformed Therapeutic Facilitation, Gabriela (Clinic 1 Occupational Therapist)

Gabriela conveyed the uncertainty she experienced at the onset of the pandemic. She remembered how clients started canceling their appointments during the week leading up to Los Angeles County's ‘safer at home’ orders. Soon, she and the other Clinic 1 therapists were left with almost no appointments and an empty clinic. She recounted:

So, that last Friday, [March] 13th, forever remembered, memorialized, a lot of us were kind of just sitting around because most of our kids cancelled […] And the following Monday we were told that everyone was staying home and that we were trying to figure out how to continue to see our kids and support our families, but that there would be a long pause until that was figured out.

Gabriela and the other employees realized they would have to either shut down their services or adapt. The Clinic 1 administrative team proposed telehealth as a solution. Initially, Gabriela was hesitant to make the transition: “Doing telehealth was completely foreign to most of us. And my impression of telehealth was like, ‘Oh, I don't want to do that, I want to see kids in person.'” Despite her lack of experience and her preference for in-person connection, Gabriela agreed to telehealth, as it was her sole option for providing occupational therapy to clients and their families. “But when you're faced with the choice of not seeing any kids at all or doing telehealth,” she recalled, “and not being able to support the families that you want to support, it's just like, ‘Alright, let's do it.'”

Gabriela described the shift as initially chaotic: “That first month of telehealth was kind of a scramble, of like, ‘Okay, how's this going to work?’” She further encountered barriers adapting certain interventions to telehealth contexts. “For my occupational therapy [versus feeding therapy] kids, it was a little bit more difficult, because I was used to being so much more hands-on, like in the gym or with any sort of, like, fine motor activities,” she said. “I was there to be a little bit more hands-on, or at least model for the parent.” Despite these challenges, Gabriela felt that her previous experience with parent coaching was a key facilitator in her transition. She noted the central import of parent coaching to the telehealth context:

So, and I've always done more, like – or at least try to do more, of a parent coaching style, which actually is harder in person because I like to be hands-on and I like to model. Whereas in telehealth, you're forced to coach. Like, you *have* to because you're not there.

Gabriela described the process of conducting telehealth as *refining* her existing parent coaching skills. She expressed that she developed better communication skills in breaking down her therapeutic processes in ways that parents could understand: “So it's, like, really honing in on the way I'm describing things, really honing in on narrating in understandable terms, […] so that parents are seeing that, as well, and are kind of taking note.” In turn, her evolving coaching expertise improved her ability to guide parents to conduct hands-on interventions. Gabriela noted that a positive outcome of her detailed coaching was that she empowered family members to learn therapeutic skills and thereby better independently support autistic clients. She shared an example of working with a client's grandmother:

I see one little guy who probably falls in the ‘moderate to severe’ [autism] category. I mainly work with his grandma, and she's actually been really great. And I really have to be a little bit more pointed and, like, almost talk to her as if I was talking to an [occupational therapy] student, explaining certain clinical observations, but in terms that necessarily don't have to be, like, professional terms. Like, more understandable terms. […] I think that was helpful for the grandparent, hearing those, kind of, specific strategies so that she can generalize that to other activities that she might try to do with him.

Gabriela further identified her clinic's focus on emotional co-regulation as beneficial. She stated that co-regulation skills were foundational to *both* her in-person and her virtual therapeutic practice. However, she found that these skills were at times particularly applicable during COVID-era telehealth, as autistic clients and parents were often overwhelmed by the circumstances of virtual therapy and of the pandemic itself. She emphasized the necessity of employing co-regulation techniques to bring clients and parents to a calm baseline before they could work on telehealth occupational therapy goals:

It all goes back to regulation, which is what we would work on in clinic and at home, too. Supporting families in figuring out how to help everyone co-regulate together so that we do have room to work on some of the targeted goals. Are we starting at a foundation of regulation? These things are important. Which, regardless of environmental context, that's what we're working on, first and foremost.

Gabriela identified her DIR®/Floortime™ background as a differentiator between her experiences and those of other practitioners. She knew other occupational therapists for whom the transition to telehealth was much more difficult, as they did not have prior parent coaching experience. Of her transition, she summed:

So, I think it's been successful. At least for us, Clinic 1. […] But, like I said, I think because we already established an ongoing routine of parent involvement, it was an easier transition. It wasn't as jarring as it was for a lot of my colleagues who are, like, in school-based or things like that.

Overall, Gabriela described her adjustment to telehealth as successful and transformative in terms of her therapeutic practice. She first engaged in virtual delivery only out of necessity, amidst the crisis of her clinic shutting down their in-person services due to COVID-19. However, she came to see the change as an opportunity to refine her coaching abilities and promote parental empowerment. She identified her application of previously-developed skills (e.g., parent coaching and emotional co-regulation) as facilitating the transition with greater ease relative to other therapists. She further felt that these skills were integral towards her clients' and their families' achievement of therapeutic benefits. Gabriela's experience thus exemplifies how specific therapeutic factors (e.g., model use and associated skills) differently impacted participants' transition to telehealth and shaped interventions during the pandemic.

#### Transformed Ways-of-Being-Together

The shift to telehealth transformed parents' and occupational therapists' ways-of-being-together, as they spent more time communicating and collaborating than they had prior to the pandemic. Therapists shared that parents were more open and vulnerable in the context of the pandemic and having to develop comfort with being ‘seen’ in their homes, interacting with their children under stressful circumstances. This vulnerability, therapists observed, helped to build a stronger relationship between the two parties, particularly as they were both experiencing the ‘shared trauma’ of the early pandemic days. Miriam (Clinic 1 occupational therapist) shared her process of learning to talk to parents about their mental health:

I think I feel a lot more comfortable talking to parents about their own mental health and learning style. […] I think, especially because we've all been through this shared pandemic, trauma experience, whatever you want to call it, it's something that we're all going through together.

However, the shift in therapist-parent dynamics and increased togetherness during telehealth was not always experienced as beneficial. Mila (Clinic 2 parent) expressed that, while she appreciated the coaching she received, she felt insecure from being constantly observed by therapists via Zoom during sessions. She clarified that her feelings had nothing to do with her therapists' skills or presence, but rather the context of telehealth itself, as she could not escape the ‘gaze’ of the camera. For her, being ‘seen’ in her home created an uncomfortable level of vulnerability and stress. She described:

I mean, I kind of had an idea [what telehealth] was going to be. […] But, it wasn't, like – you know, it was just a bit challenging with strangers, you know. Or, not strangers, but, like, you know, with people that you don't want, you know, to see you in a camera. […] Yeah, it was a bit – you know, just the insecurities that, like, you know, think for yourself and, like, you probably overthink yourself. Well, what is he thinking, or all of that stuff, you know.

The pandemic and telehealth also transformed parents' ways-of-being-together with their autistic children. During the pandemic, parents spent more time with their children than they did under normal circumstances. Some of this additional togetherness was due to the context of the pandemic itself, as families were confined to their homes. Additionally, as noted prior, telehealth necessitated more parental time and involvement with their autistic children to help facilitate therapy. Several parents expressed that the resultant increased togetherness deepened their parent-child relationships. Zoe (Clinic 2 parent) shared how her dynamic with her son, Charles, changed throughout the course of telehealth. As she spent time with Charles in therapy, with each of them learning new skills, she developed a new sense of shared accomplishment with her son:

And he actually progressed a little bit when we were doing the telework. […] I didn't know, I didn't believe that we could actually, like actually meet the goal. Like, you know, the blowing [goal]. Because before he couldn't really – that's it, he can't even blow a candle or can't even hold a … He can't even hold a scissor. And we actually finished those goals while teleworking. We were actually, and we were doing assessments like *hey*, we did that! *We actually did that.*

However, parents also experienced stressors and burnout from the cumulative demands that telehealth placed on their schedules and energy. They felt challenged to help their autistic children adjust to virtual delivery and to keep them engaged during sessions, particularly on top of additional responsibilities such as working and caring for other children. Zoe noted that being with Charles in telehealth took time from her other tasks: “And it's kind of like stressful, too, because I'm like, oh my God, I can't do anything because I have to sit there with him.”

Overall, parents found increased togetherness with their children in the context of telehealth delivery to be stressful *and* beneficial to varying degrees. We thus present the following two vignettes to impart the differing ‘poles’ of parental experiences related to the theme. Both Alexis (Clinic 1 parent, Vignette #2) and Mila (Clinic 2 parent, Vignette #3) experienced the shift to telehealth as transforming their ways-of-being-together with their autistic children. However, Alexis experienced this transformation as largely *beneficial* through increasing her time spent together with her child, whereas Mila found it to be mostly *stressful* through depleting her family's time spent apart. In detailing Alexis' and Mila's stories, we explore their differing perspectives on the advantages and limitations of telehealth-delivered occupational therapy for autistic children.

#### Vignette #2: Transformed Ways-of-Being Together, Alexis (Clinic 1 Parent)

Prior to the pandemic, Alexis had struggled to feel connected to her son, Liam. She expressed that she and her husband had always loved and tried to bond with him. She had, however, perceived a lack of reciprocity from Liam in turn:

When he was born, the moment my husband and I laid eyes on him, there was just *so* much love. I mean, we just loved him from day one. […] But I can't say that Liam had it for us. And that's not to be mean or anything, it's just, I don't think he knew how to do that or anything. […] He didn't have any attachment or anything like that.

COVID-19 brought many stressors for Alexis and her family, but also new opportunities for them to connect. Under ‘stay at home’ orders, Alexis and her husband were working remotely full time as teachers while also caring for Liam and supporting his remote participation in his educational and therapeutic programs. Despite managing these complex demands, she said that participating in telehealth-delivered occupational therapy was beneficial and transformative, as it allowed her to learn “how to be a kid again, and how to play with [Liam], and how to understand him.” She attributed the effectiveness of her telehealth sessions largely to her therapist, Gabriela, who coached her not only through typical occupational therapy activities but also through moments of Liam becoming frustrated or dysregulated during the sessions:

And [Liam] would just lose his temper, he would just have a tantrum. And Gabriela's advice was to narrate [his emotions], and to *feel* with him. […] So I reluctantly got a little dramatic with him. And by golly, he gave me a kiss! He was *super* excited. Like, you could tell, like, all of a sudden, “Mommy *gets* me.”

Gabriela guided Alexis through difficult moments of Liam's dysregulation, giving her not only strategies but altogether different ways of framing his behavior and experiences, resulting in transformed ways-of-being-together for the mother and child. With Gabriela's coaching, Alexis went from feeling confused by Liam's behaviors to gaining new understandings of his emotions and perspectives: “Sometimes you're like, ‘This is ridiculous. Why are we having a tantrum over this?’ But then you put yourself in their shoes and how they view the world, and it's *really* powerful.” Through their increased togetherness and improved emotional co-regulation, Alexis observed a marked difference in Liam's displays of affection: “And during the course of the pandemic, he's very attached to us now. […] You can tell, he *loves* Mommy and Daddy.” Alexis further witnessed Liam seeming to want to spend time together with her and her husband. She described how, for the first time, Liam initiated including them in his play and self-expression:

And now what he's doing – and I think this is remarkable. I've never seen… He will come, he gathers us up at home. So he comes, he grabs our hand, and he pulls us into a bedroom. […] He does flips and rolls on the bed and does twirling and like this big, huge, elaborate thing. And if you move from your spot, he gets mad. […] And then we'll just start clapping and cheering and be like, “Woo!” And, by golly, the kid *smiles* and *enjoys* that praise.

For Alexis, the pandemic represented a turning point in her relationship with Liam as his prior indications of low attachment transformed into reciprocal parent-child bonding, with telehealth-delivered occupational therapy serving as a driving force for this change. “He's coming out of his shell,” Alexis expressed, “[…] We're at home in the environment, and we're getting the coaching. And that's really beneficial. And, like I've already said, the bonding has just – it's been tremendous.” Telehealth provided the mother and child with opportunities for not only increased *quantity* in the spent time with each other, but a deeper, more reciprocal *quality* in how the pair related. Ultimately, Alexis' and Liam's mutual therapeutic engagement profoundly transformed their ways-of-being-together in a positive direction.

#### Vignette #3: Transformed Ways-of-Being-Together, Mila (Clinic 2 Parent)

Like Alexis, Mila's experiences of telehealth transformed her ways-of-being-together with her family. However, for her, the increased togetherness presented more stressors than advantages. Mila felt that the shift to telehealth placed high demands on her and her autistic son, Enzio. Per her report, Enzio initially struggled to recognize and listen to his occupational therapist, Lisa, despite the strong rapport that the two had built during their in-person sessions. She felt that the shift to telehealth was detrimental to their therapeutic relationship. She further observed Enzio having negative reactions to the change in his therapy routine: “You know, [telehealth] was just so different for him, it maybe scared him, I don't know.” She noted that Enzio had difficulty sitting still and paying attention in telehealth sessions, and she struggled to keep him calm and engaged while assisting with interventions via Lisa's coaching. In addition to helping Enzio, Mila had to simultaneously care for her two-year-old daughter, Tina. She felt overwhelmed managing both children during sessions. When asked about how she felt regarding the transition to telehealth, Mila replied:

I didn't really like it, because I know Enzio. So, yeah, I knew, yeah, I wasn't going to like it. For a lot of reasons, not just because of Enzio. Because at the time I had my daughter and she was still a baby, so she needed my attention. […] And so, it's all over crying because Enzio does not want to sit down, you know, at all. He doesn't want to pay attention, he doesn't want to sit down. Tina is the one that wants to be in the Zoom, and she's not getting services. So, we go back to, you know, it's just so – it was just back and forth, being with – Tina wanted to be on Zoom and Enzio [didn't].

She noted that Enzio seemed to do better in a clinic setting, where he was in a separate space from his sister, was less distracted, and could receive one-on-one, face-to-face attention from Lisa. Further, as touched on prior, Mila appreciated that in-person services offered more flexibility in letting her opt to attend versus requiring her presence. She felt as if her presence interfered with sessions and Enzio's dynamic with Lisa:

Because, like, you know, I felt like if I were to be around them, I probably would have been a distraction. Or, you know, it was just like the whole one-on-one thing, you know. Or, maybe I would have, like, undermined her on some – you know, without me thinking or something. I just wanted them to just have their own time together.

In sum, Mila expressed that *time spent apart* during therapy was a valuable resource that helped ensure that she and her children could meet their needs. When the pandemic hit and telehealth-delivered occupational therapy was implemented, Mila and her family experienced erosions to their time spent apart. For them, telehealth demanded a high-pressure, overwhelming amount of togetherness during sessions that prohibited Enzio and Tina from receiving the individual attention that each child sought. Mila, consequently, was tasked with managing their competing demands, while also acting as a hands-on therapeutic facilitator. Mila did express that she experienced some benefits from telehealth occupational therapy in terms of increased togetherness, such as growing in her abilities to multi-task her children's needs: “I've learned to be more tolerable with both kids, juggling them around, you know.” In Mila's case, however, the benefits of telehealth-delivered occupational therapy during the pandemic did not outweigh the disadvantages. Her story thus illustrates how increased togetherness in the context of telehealth-delivered occupational therapy during COVID-19 could have had primarily detrimental, stress-inducing consequences for some families.

### Personal Transformation

In addition to transformed therapeutic facilitation and ways-of-being-together, parents and therapists shared experiences of transformative *personal* growth, wherein they realized new understandings about themselves, their relationships, and others. These realizations often dramatically shifted their prior ways of thinking. Parents' personal transformations generally involved realizing novel capabilities and potential in themselves and in their children.

Fernando (Clinic 2 parent) talked about how Luis' progress in telehealth-delivered occupational therapy “surprised” him. Luis' developmental challenges, notably his speech delay, initially made it more difficult for Fernando to understand his son's capacities. Fernando described how, before telehealth, he sought to do things *for* Luis: “You know, because, like, I don't want to – yeah, I'm always trying to support him, and I always want, you know, to kind of ease his – make things as easy as possible.” While Fernando sought to protect his son, he felt as if his efforts might have, to some extent, prevented Luis from being challenged enough to grow. His experience with telehealth was eye-opening:

I think this taught me that, like, [Luis is] actually much stronger than you think. […] It's been great to see him – because he doesn't tell us, like, what he learned today in class, or other things, because his speech is delayed, so he's not there yet. But being able to see his process during teletherapy has been really informative, and also rewarding. Because I kind of feel like I'm seeing how his mind works, how he puts things together and how he makes connections between things in a way that I wouldn't get to see if it was just us at home, you know, playing or whatever.

Telehealth revealed more of Luis' autonomy and abilities to Fernando, ultimately transforming the father's understanding of his son's potential.

For Alexis (Clinic 1 parent), the shift to telehealth afforded an *equalizing* opportunity to discover her own agency. Alexis shared how she felt empowered by the change: “And I think, in a way, [telehealth] was very empowering because we weren't relying on the therapist anymore as the one that's engaged with the kid and everything like that. Now the therapist is forced to literally coach us and to tell us what to do and give us ideas.” Via coaching, Alexis acquired skills which are typically more exclusive to occupational therapy professionals within in-person pediatric clinic settings. Without having to rely as much on her occupational therapist to conduct interventions, Alexis developed newfound capabilities that allowed her to bond with Liam in ways that she had not been able to before. From this bonding, Alexis gained a deeper understanding as to the import of the parent-child relationship. “I didn't realize how important it was,” she noted, “to earn their trust or their love and affection, and prove to them that you're their safety net, and that you're always going to be there for them.” Alexis' personal growth, shaped by the access to knowledge that telehealth afforded her, empowered her to transform her connection with Liam in turn.

Similar to Alexis, Zoe (Clinic 2 parent) also developed increased feelings of competence and confidence in supporting her child, Charles. She detailed how her occupational therapist, Lisa, would suggest and model new skills for her that helped Charles to learn. As she learned and increasingly helped facilitate these skills, she witnessed Charles develop greater agency: “[Lisa] would tell [me], like, ‘Hey Zoe, try doing this,’ because she can't really be here to like show it to Charles. […] So, you know, you learn creative ways to do certain things with him. Yeah, and it works, because he can eat by himself now.”

Like Fernando, Zoe witnessed her child gradually progress in ways that she previously did not know were possible for him. She described how Charles would excitedly show off his increased independence to her: “Ironically, when we did the teleworking thing, he actually learned the work, like, ‘I can do it by myself. I don't need help.' So, that was a positive thing. Because he was like, […] ‘I can do it, Mommy.'” Via telehealth-delivered occupational therapy, Zoe thus transformed in her understanding of what both she *and* Charles were capable of. Charles' therapeutic gains were facilitated by her personal growth and her collaboration with the occupational therapist to support his learning.

Therapists' personal growth intertwined with their professional roles, as their internal journeys often translated to their clinical practice. Some therapists, like Lisa (Clinic 2 occupational therapist), were not used to practicing with a parent coaching model prior to the pandemic. Lisa experienced a subsequent initial adjustment from conducting intervention activities herself to verbally guiding parents through carrying them out. While Lisa described this adjustment as stressful, she also expressed that it helped her grow as a therapist:

I was really nervous about it. But the interesting thing is like it was almost like this silver lining, because we were thrown into it. And I felt like I actually became better, because I had to learn how to like talk the parent through something. Which was so different from just doing it myself. So it was really hard, and by no means was I good at it, like at all, but I feel like I learned like what works and what doesn't work.

Jenna (Clinic 1 occupational therapist), who described herself as a new practitioner, recounted how her experience with telehealth increased her confidence in communicating with families. “So, I think – because I am, I feel like I'm still a very new clinician,” she recalled, “[telehealth] was definitely like a growing process of feeling more confident in being able to explain things to families. And now, I feel like that confidence is definitely at a much very different level.” Meanwhile, Miriam (Clinic 1 occupational therapist), who had many years of clinical expertise, recounts how her experience with telehealth allowed her to reassess her role as the ‘expert’ and appreciate the role parents have as experts in their children's lives:

You know, with all of my work and my training, we always know that the parent is the expert on their child. But I think, with the power dynamic of being the clinical expert and being paid to help, there's all these layers of feeling like on some level I know best…But I think it's been really wonderful to see and to be surprised - it's been humbling to be surprised at how well these parents know their kids.

Therapists, in sum, found the transition to telehealth to be challenging, but also a process of adaptive development. They gained new skills and deepened appreciation for parent coaching and the importance of supporting families. Some, like Miriam, reconsidered their roles as ‘experts’ during sessions as they came to increasingly value parental insights and involvement. Others came to discover and better appreciate their own clinical expertise. The shift to telehealth-based coaching thus provided occupational therapists with unique opportunities for both personal and professional transformation.

### Reimagining Therapy

The shift to parent coaching had profoundly transformative implications for participants' experiences during the pandemic. However, the telehealth transition necessitated that therapists *reimagine* many additional therapeutic nuances that were finer-grained and more specific to clients' environments and daily routines. Therapists' goals (e.g., supporting client feeding) and interventions often remained consistent from in-person to virtual delivery. While they did not completely *transform* these goals and interventions for telehealth, in terms of their core intents and practices, they *re-imagined* how they could be implemented effectively in the telehealth context. To this end, therapists modified environmental factors and adjusted how specific intervention components were carried out in clients' homes. While these alterations were often granular *in isolation*, they could amount to big-picture results and even bear ramifications as to the nature of occupational therapy.

This reimagination, notably, presented deeper existential implications for what occupational therapy could be. Many had been trained, even if implicitly, to view pediatric occupational therapy practice as contingent on in-person norms (i.e., delivered in a therapy gym with specialized equipment and materials). The transition to telehealth brought these assumptions into question as therapists realized that, via their adaptations, they could reimagine and expand occupational therapy beyond the clinic setting. Miriam (Clinic 1 occupational therapist) described how her experience with telehealth-delivered occupational therapy generated an opportunity to develop a different understanding of therapeutic possibilities:

The switch to telehealth at first felt like, “Oh, my gosh. How are we going to do that? […] There's no way we can help this child when we don't have our zip line or our ball pit (laughs), or when […] I can't touch his mouth and feel the tone.” But then, the flipside is, if it had to be that specialized, how much were the parents really involved, right? And how much was that work going to be translating, ultimately? So, I think a lot of my initial reaction of, “Oh, this is going to be so hard,” pretty quickly shifted to, “Well, this is going to be different, and it'll be hard in its own way, but it's not impossible.”

Miriam described a clever adaptation to telehealth therapy sessions for clients who got easily overstimulated by the extra “noise” of the therapist on Zoom. She said:

There are a couple families where I'll purposely [turn off] my video and the parent will have a Bluetooth headset. And they're just feeding the child. And they pop me up, and I'll just kind of be whispering things in the parent's ear, without the child seeing that it's there, so that it feels more like, “Oh, Mom's just on the phone.” But we're interacting together. […] So those are some things that evolved over time. They didn't look that beautiful when we started. [Initially] I was kind of like, fumbling around and trying to figure out what's the most helpful. And what are we just trying to force because we think it has to look a certain way?

Miriam's “fumbling around” for a solution was prompted by a client, an autistic child who was new to her, and to Clinic 1, during the pandemic. Because the family had not had in-person sessions, they were not yet familiar with the clinic's child-led, play-based style. Miriam noticed, during the first few sessions, that the child was extremely dysregulated during the first part of the session. She said:

I had no idea, but the whole 30 minutes before our session, [the mom] was working *so* hard to try to [have] this kid seated in a highchair right when the Zoom video would be on, so that I could see him eat. And I had *no idea* that was happening. […] So the first 15 minutes [of the session] was working on just helping him feel calm and relaxed, and trying to get him to stop reaching for the phone that had my face, and throwing it across the room, or smashing it in his mashed potatoes, and then all I was seeing was white, blurry clouds for a little bit, and feeling like, “Wow. Okay. […] Clearly sitting in his highchair and engaging is *so* hard,” not knowing that he was on the tail end of like a half-hour struggle just to sit in the highchair, which I never said was a requirement. But I also never said it *wasn't* a requirement. […]

It took a couple sessions before I was starting to feel like, “Hmm. I feel like I'm missing something.” And there were a couple sessions of me trying to ask gently, “So, is this how he normally is during lunch? What do you normally do? Just help support him like you usually would.” And really not feeling like I was getting the answer to the question I was trying to ask. So, then I finally got a little more direct, like, “Hey, quick question. Is he in the highchair because this is normally the time? Or is it because you're trying to be ready for therapy?” And she was like, “Oh. I'm getting him ready for therapy.” And then that opened it up to this whole discussion of like, “What does it look like to get him in the highchair?” And she's like, “Oh, you have no idea. The first five seconds when we start the Zoom call, and our video is off, it's because I'm wiping the sweat off of my forehead, because we've been working so hard. And I'm wiping his tears away so that we can be ready for therapy, because we want him to learn.” […] I felt so horrible, but I was also so glad that we were able to get to that point of really open conversation.

Once Miriam realized that the mother had assumed that the child must be seated in his highchair in order to have a successful therapy session, Miriam was able to recruit the mother in reimagining the session together. Miriam assured the mother that she wanted the child to eat as he naturally did; as they talked about what mealtimes typically looked like, the mother told Miriam, “I don't know why this is so hard for him. I talk to my sister on the phone all the time when we eat dinner." Miriam told the interviewer:

And then we realized, like, “*Oh*, it's not FaceTime. It's just a phone call, and she has her AirPods in! So it's not this extra thing that is visibly taking her attention.” […] So, then that's how it evolved into her wearing headphones, so that I could talk directly to her and he wouldn't hear me, but that she could still respond to me. And that was okay for him. So, that's how we ended up doing things.

For many therapists like Miriam, telehealth-delivered occupational therapy initiated a shift in common-sense assumptions about occupational therapy norms. In the early stages of the switch to telehealth, they often felt pressure to convey a sense of control and keep telehealth interventions as close to in-person practice as possible. However, they soon began to reconsider their therapeutic expectations. Without access to clinic resources, and with an increased amount of parent-therapist collaboration, therapists became more flexible towards unexpected circumstances and problem solving in new ways. Gabriela (Clinic 1 occupational therapist) described how her transition to telehealth required creative adaptations to situate her feeding interventions in families' homes and routines:

For my feeding clients, joining into a naturalistic feeding or mealtime context was pretty easy [during telehealth sessions]. They would just have to put the device on the table and I would do a lot of observation, and I would just kind of join whatever snack or mealtime that was happening at that time. It was a little bit difficult in terms of clinical observation because I couldn't get in and see if the child was swallowing, I couldn't get much details about how their mouth was moving. So I would have to rely a lot on parent report or ask a lot more questions than I would have if we were in person, because I would just be able to observe and see it.

While the core components of Gabriela's feeding interventions (e.g., assessing oral motor function) remained the same from in-person to telehealth, she had to reimagine how these components were to be carried out in the client's home. This reimagination process included her considering how to position the camera to facilitate clinical observation, as well as relying on parent report. However, Gabriela noted the relative ease of her transition and the benefits of conducting telehealth in clients' natural environments. This latter aspect was especially appreciated by occupational therapists generally, with several expressing that a prime advantage of telehealth was their ability to observe and apply interventions directly to clients' natural environments. “I think telehealth really helped to get an insight into what's the situation at home in the most, like, you know, comfortable way for the families,” Hannah (Clinic 3 occupational therapist) noted, “and support them that way so that they can turn some things around in the best way.” Hannah described how gaining insights into the home environment helped her reimagine her feeding interventions with a particular client:

And when I went in the home environment, I see that [the client is] usually running the show. And her highchair is in front of the TV. […] So the TV has to be on, so there's no active participation in mealtime. And the mom is like struggling, like she's trying to like hide the food, and like distract her [to get her to] eat it. Whereas in the clinic, I'm trying to like have her actively engaged, but that's not her daily routine.

With this new information, Hannah encouraged the client's parent to gradually turn the TV off during her regular mealtimes and become more involved in feeding. The parent agreed to do so. Hannah described this adjustment to the family's normal routine as progressing the client's independence: “So now like the child is, you know, more actively participating, even in sessions with me. Picking up food by herself. Trying to use the spoon. […] It was like a huge shift, like just turning off the TV was big.” Hannah's view into the client's home environment during telehealth facilitated her discovering a barrier to mealtime participation and a therapeutic solution that would not have been apparent in the clinic setting.

Occupational therapists thus discovered highly creative ways to reimagine interventions for telehealth. They leveraged materials and resources that were readily available to families and embedded activities into clients' typical routines. They also utilized technological strategies; as previously described, some coached parents via Bluetooth and turned off cameras to reduce overwhelming sensory stimuli, and others used Zoom's chat features and whiteboard and recorded videos to model strategies for parents.

Therapists' reimagination exemplified how seemingly minor adjustments—such as implementing text-based instructions or having a client's parent turn off the TV during regular mealtimes—could have significant therapeutic impacts. The delivery medium of telehealth provided unique opportunities to tailor their recommendations to families' everyday habits and environments to maximize their impact. Further, therapists could compare the impacts of their in-person versus virtual interventions. Sophie (Clinic 1 administrator and occupational therapist) reported how some considered telehealth to be a ‘return’ to occupational therapy fundamentals, via emphasizing the centrality of occupation and the translation of interventions to natural client contexts:

And over time, what I would hear from the [occupational therapists] is, “Whew, I feel like maybe I'm a better [occupational therapist] now that I'm out of the sensory gym. I feel like I'm more hitting the core of our professional identity, doing meaningful occupation, when I don't get to rely on having a ball bit and a zipline.” The occupation of play is very important, and we will all continue to say that it's incredibly important, but when I didn't get to rely on the zipline, what do you do instead? Oh, you do activities at home. So we were cooking together, and we were playing in their own physical space. And we were doing the things that, for years and years and years, we've talked about in early intervention: the least restrictive environment and a natural environment – and the *power* of that. Now, we have that for every single kid across the board. And that was a big shift.

Through their telehealth experiences, therapists implemented numerous adaptations and discovered both an expanded vision of occupational therapy's possibilities and a return to the fundamentals of occupational therapy practice. The sudden and drastic changes to routine, in-clinic therapy practices sparked by the pandemic created new opportunities for a different kind of growth, one in which the nature of therapy itself, could be reimagined.

In this section, we have primarily focused thus far on *occupational therapists*' experiences of reimagining therapy. We now conclude with Vignette #4 to provide an administrator's perspective on this theme. Prior to the pandemic, clinic administration was set up for in-person services. Like occupational therapists, administrators were also forced to rapidly adjust to telehealth as a new mode of service delivery. Sophie's story below illustrates her journey to adapt and to reimagine the norms of occupational therapy provision, from the managerial end.

#### Vignette #4: Reimagining Therapy, Sophie (Clinic 1 Administrator/Occupational Therapist)

As both an occupational therapist and a clinic administrator at Clinic 1, Sophie was not only responsible for providing services to her clients, but also for protecting the livelihood and well-being of her entire team during the pandemic. She recalled how, in January 2020, some employees in her workplace who had family members who were impacted by the pandemic before it reached the United States approached her to express concern and advocate for employees and families to wear masks, even before the government mandated those protocols. To Sophie, these warnings initially seemed farfetched and a big ask of their clients. However, in hindsight, she said they were a ‘foreshadowing’ of what was to come.

By early March, the clinic administrators were becoming concerned about the gradual decline in clinic attendance. Measures had been put in place to ensure that clinicians were following health protocols, washing hands regularly, sanitizing rooms, and cleaning floors. However, by the second week of March, the Clinic 1 operations manager could not source many cleaning supplies due to the global shortage. In addition to the decline in attendance, clinicians also began expressing their fears about coming into the clinic and going into the homes of families.

Sophie and other administrators faced incredibly tough decisions about how to handle the ongoing provision of services. They would not be able to pay their clinicians should their funding reduce due to a change in service delivery. Therefore, they contemplated putting everyone on temporary leave or furlough. However, they were conflicted by the fact that a number of staff members would be adversely impacted if they suddenly lost their income. Sophie and the other administrators opted to lay everyone off for a two-week period so that the clinicians could access unemployment and have enough income to pay rent and cover the costs of student loans. They further decided to roll-out a six-month transition plan before the clinic would have to close. Sophie expressed that these decisions were challenging for her both as a therapist and as an administrator who was “in the position to have to make those choices about the financial livelihood of our clinical team.”

However, Sophie and her team discovered that their funding sources would allow, and in some cases even encouraged, them to provide services via telehealth. In what Sophie described as “a leap of faith,” she and her team consequently decided to adapt to telehealth, despite Clinic 1 only being set up for in-person services. Once Sophie's team had approval to deliver telehealth, they reinstated their clinicians' caseloads. It took some time for Sophie and her team to figure out which platforms were most effective and HIPAA-compliant for telehealth, and for some clinicians to adjust to using the various technologies. The administration also contacted families to discuss the new arrangements. A few families did not want to try telehealth (i.e., largely private pay families); but most families were open to trying it. Sophie developed mutually supportive dynamics with these families as they navigated the telehealth transition together: “I remember some of the parents that I worked with, those moms were like, you know, ‘How are *you* doing?’ And I'm like, ‘*Me*?’ I was so fragile and so raw.” Of the transition, she summarized: “It was really hard. But we just kind of dove in and did it.”

Sophie faced manifold barriers to keep her staff employed; adjust service delivery to a virtual format; navigate funding and constant situational unpredictability; and partner with families to ensure ongoing care. Further, she navigated additive pressures from managing dual roles as therapist and administrative staff. Despite these pressures and barriers, Sophie continuously worked to make telehealth viable for her Clinic 1 team and the families they served. Therapists' re-imagining of interventions at the *clinical* level were facilitated by Sophie and other administrators' re-imagination of service provision at the *infrastructural* level.

### Going Forward

Our final theme reflects participants' perspectives on their eventual return to clinic-based care and what therapy should involve going forward from COVID-19. Some parents and therapists raised concerns about the transition back to in-person services when the restrictions of the pandemic lifted. Gabriela (Clinic 1 occupational therapist) shared her apprehension about the translation of a client's skills gained during telehealth. “Just because he's met those goals at home in that context,” she observed, “doesn't necessarily mean that he'll carry that over once his regulation is challenged when you have to go to different places and have to go through more of those transitions, like environmental transitions.” Gabriela also shared that some of her autistic clients seemed to do better in telehealth than they had in the clinic: “And then also, it's been interesting for some kids, telehealth has actually been easier for them because their regulation hasn't been challenged because they're home, they're in their comfortable place.” Miriam (Clinic 1 occupational therapist) was concerned about preserving the good that telehealth had brought about:

Of course, there are some things that we'll be able to do a lot more easily in person. But at the same time, how do we maintain all of these other beautiful things that have happened because of telehealth? And how do we protect these parents and their more recent sense of confidence or their sense of being the expert? We don't want to lose that just because it's back in person and they're coming to us. So I think that's something that is an ongoing conversation in our department.

Parents shared how telehealth could be beneficial for themselves and their children. Zoe (Clinic 2 parent) expressed how her son, Charles, anticipated his Zoom sessions: “For the telework, it was actually positive for him too. Because he was always looking forward to it because he's, you know, he can play with certain things. So, it was positive for him.” Envisioning the future of telehealth, some parents indicated an interest in continuing with virtual or hybrid services. Alexis (Clinic 1 parent), who had no prior experience with feeding therapy before telehealth, expressed that she did not feel an immediate need to transition to in-person services. She had found value in conducting some intervention activities, such as feeding, over Zoom, where they could be situated in her home environment:

I'm glad that we had this journey. And the other day, I was talking to Gabriela, and I told her, “I wonder if they're going to have feeding therapy through Zoom.” Because something like that…I mean, obviously, I've never really done feeding therapy in the clinic, but I would imagine it's really hard to bring all the food all the time and stuff, whereas it's really nice to just have her on Zoom. We can prepare everything in the kitchen. […] I guess that's one therapy that I wouldn't mind necessarily - like, I wouldn't mind staying on Zoom for because it is really helpful, you know?

Zoe suggested that a policy be implemented that allowed for service continuity in the event of a pandemic. “If a pandemic strikes, services shouldn't stop,” Zoe contended, “because I know a lot of parents struggled because they had to fight with their insurance company, because the insurance company would not provide those services.”

Mila (Clinic 2 parent), conversely, believed that telehealth was less effective than in-person occupational therapy for herself and her family. She observed that her son Enzio did not make the same progress in a virtual setting that he might have made in the clinic: “I really didn't see any improvement in Enzio's case, you know, when it came to Zoom.” Further, she felt overwhelmed by the demands that telehealth placed upon her compared to in-person occupational therapy. She was grateful for the return to in-person services. However, she noted that her generally negative telehealth experiences did not necessarily apply to other families: “I can't speak for all autistic children or parents that have children with autistic-you know, with autistic children, but it's really hard, in my experience, to have Zoom as something that can be used for Enzio."

Finally, Emily (Clinic 2 administrator and occupational therapist) asserted that telehealth could continue to be a “viable alternative” to in-person services post-pandemic, as it heightens the creativity of therapists and is “another great way to help approach disparities and access.” She described how, prior to the pandemic, research had examined the efficacy of telehealth-delivered occupational therapy, but “there's been a lot of roadblocks in terms of making that something that is accessible from a funding agency perspective.” She expressed a desire for the pandemic experience to open up new funding, research, and clinical possibilities for ongoing telehealth-delivered occupational therapy moving forward:

I'm hoping and we're hoping that this is a turning point in allowing for this to be something that is really considered research based, and viable, and meaningful, and family-centered, and child-centered, to be able to help the people that we're hoping to help.

## Discussion

In this qualitative study, we examined how occupational therapists, clinic administrators, and parents of autistic children experienced the shift to telehealth-delivered occupational therapy during the COVID-19 pandemic. We found that this transition required participants to *reimagine* the norms of occupational therapy practice and to *transform* their therapeutic facilitation, ways-of-being-together, and personal journeys, with both beneficial and detrimental aspects. These findings build upon our previous study (Angell et al., 2023), which identified facilitators and barriers to telehealth occupational therapy implementation, as well as positive and negative therapeutic outcomes. They additionally align with other research conducted on occupational therapy during COVID-era telehealth. Similar to Rosenfeld and Brooks (2023), we identified that occupational therapists' therapeutic reimagination required substantial creativity to adapt to virtual delivery, that parents often became ‘therapy assistants', and that interventions were often more accommodating to individual client needs and transferable to the home environment. Echoing Lamash (2023), our findings suggest that telehealth-delivered occupational therapy might be particularly strengths-based, as therapists actively collaborated with parents rather than assuming a strictly directive, ‘expert’ role. Like Sleight et al. (2023), we found that participants' views on telehealth's effectiveness varied based on the interventions employed. Specifically, our participants experienced telehealth-delivered occupational therapy as conducive for parent coaching, consistent with previous findings (Camden & Silva, 2021; Little et al., 2023; Rosenfeld & Brooks, 2023). Finally, we explored participants' preferences for telehealth versus in-person services *going forward* from the COVID-19 pandemic, reflecting a broader body of work on the long-term viability of virtual occupational therapy (Angell et al., 2023).

A key limitation of our study is that we did not elicit the perspectives of autistic children receiving telehealth-delivered occupational therapy. There is a dearth of inquiry exploring autistic children's experiences of occupational therapy services, both virtual and in-person, and more research is needed. Further, we interviewed occupational therapists who used three therapy models, which is not reflective of the full scope of pediatric occupational therapy practice. Finally, we collected data from April to November 2021, over a year past the start of the COVID-19 pandemic in March 2020. This time elapsed between the events of the pandemic onset and data collection may have impacted the accuracy of participants' memories. However, it may have also given participants more time to reflect on their experiences and stories.

Our study employed a narrative approach, which has not yet been utilized in the occupational therapy pandemic-era telehealth literature. Our methodology thus constitutes a strength, as it enabled us to focus in on participants' stories and experiential contours of this particular time period and its highly unique circumstances. Further, we collected perspectives from multiple participant groups (occupational therapists, parents, and clinic administrators) and purposively sampled participants from three different clinics, lending greater variety to the stories elicited and enabling comparisons between how differing models subjectively translated to telehealth. We interwove our broader thematic findings with vignettes to convey narratives at the individual participant level.

These finer-grained analyses allowed us to go more in-depth into our previous data and revealed nuances not yet captured in the broader literature. Whereas other studies focused solely on healthcare providers' perspectives on telehealth (Camden & Silva, 2021; Pineda et al., 2023; Rosenfeld & Brooks, 2023), we included viewpoints from occupational therapists, parents of autistic children, and clinic administrators. While participants varied in their preferences, they generally agreed that telehealth should be an ongoing *option* for service delivery, albeit not a total replacement for in-person care. They noted the specific potential for hybrid services and the particular applicability of telehealth for certain interventions (e.g., parent coaching; supporting feeding in the home context). Despite the demand, some participants were concerned about potential challenges with continuing telehealth provision, such as whether funding mechanisms for virtual delivery would remain available after the pandemic. Our findings thus substantiate calls from other studies (e.g., Camden & Silva, 2021) for telehealth-delivered occupational therapy to remain an option for clients and their families post-pandemic. We uniquely identified support for telehealth-delivered occupational therapy options *across a consensus of multiple perspectives,* but concerns remain over barriers such as contingence on systemic resource allocation.

We also found nuances regarding participants' experiences of differing clinical models and ways of relating during virtual sessions. Families' increased togetherness during sessions could promote bonding, but could also deplete boundaries and increase burnout risk. Participants noted that DIR®/Floortime™ was *specifically* conducive for telehealth, as it prioritized coaching and emotional co-regulation to help both clients and their parents manage stressors. These findings bear implications for researchers and therapy staff considering virtual occupational therapy implementation. While our findings initially suggest the applicability of DIR®/Floortime™ to telehealth, our sample is small and limited in therapeutic scope. Researchers may benefit from conducting more in-depth, comparative analyses on how specific occupational therapy models translate to virtual contexts. Researchers could further examine the potential for differing hybrid formats to accommodate differing levels of preferred togetherness during sessions. Some parents, for example, could benefit from receiving individual virtual coaching in tandem with in-person services for their children. This hybrid option could support parents' involvement without the full pressures of therapeutic co-facilitation; promoting skill development while mitigating burnout.

Finally, our analyses illuminated distinct narrative arcs. Participants' recollections were often starkly divided between *before* COVID-19 and *after*. The pandemic and the transition to telehealth created major conflicts in their life stories. Yet, their sense-making often centered *transformation* and *reimagination* as outcomes of these conflicts. Many deepened their relationships as they spent more time together. Parents took on the challenges of therapeutic co-facilitation, which uncovered capabilities in themselves and their children in ways that often surprised them. Occupational therapists' initial struggles to adapt gradually revealed the salience of adapting interventions to natural contexts. Participants challenged the status quo of traditionally in-person occupational therapy and advocated for telehealth options. For many, the COVID-19 pandemic and the transition to telehealth represented *turning points*, or significant events, that shifted their preconceptions about themselves, their relationships, and the nature of occupational therapy itself.

## Conclusion

In our qualitative study, we found that the COVID-19-era shift to telehealth-delivered occupational therapy was a turning point for participants. Our narrative approach elucidated participant *transformation* and *reimagination* emergent from this new situation. Such findings raised deeper philosophical implications for occupational therapy practice. Challenging long-standing occupational therapy norms, we identified support for expanding delivery options beyond *strictly* in-person services. Our findings further indicate a need for comprehensive analyses on how telehealth-delivered occupational therapy could be effectively implemented moving forward. We recommend multiple perspective paradigms that elicit input from diverse ‘actors,’ including autistic children and occupational therapy clinicians, implementing a wider variety of formats and models.

## Footnotes

1 Although Alexis was recruited from Clinic 1, where her son was receiving occupational therapy at the time of data collection, this comment refers to her experience at different clinics where he had previously received services.
